# *Leishmania donovani* Infection Induces Anemia in Hamsters by Differentially Altering Erythropoiesis in Bone Marrow and Spleen

**DOI:** 10.1371/journal.pone.0059509

**Published:** 2013-03-22

**Authors:** William P. Lafuse, Ryan Story, Jocelyn Mahylis, Gaurav Gupta, Sanjay Varikuti, Heidi Steinkamp, Steve Oghumu, Abhay R. Satoskar

**Affiliations:** 1 Department of Microbial Infection and Immunity, Wexner Medical Center at the Ohio State University, Columbus, Ohio, United States of America; 2 Center for Microbial Interface Biology, Wexner Medical Center at the Ohio State University, Columbus, Ohio, United States of America; 3 Medical School, Wexner Medical Center at the Ohio State University, Columbus, Ohio, United States of America; 4 Department of Pathology, Wexner Medical Center at the Ohio State University, Columbus, Ohio, United States of America; Federal Institute for Vaccines and Biomedicines, Germany

## Abstract

*Leishmania donovani* is a parasite that causes visceral leishmaniasis by infecting and replicating in macrophages of the bone marrow, spleen, and liver. Severe anemia and leucopenia is associated with the disease. Although immune defense mechanisms against the parasite have been studied, we have a limited understanding of how *L. donovani* alters hematopoiesis. In this study, we used Syrian golden hamsters to investigate effects of *L. donovani* infection on erythropoiesis. Infection resulted in severe anemia and leucopenia by 8 weeks post-infection. Anemia was associated with increased levels of serum erythropoietin, which indicates the hamsters respond to the anemia by producing erythropoietin. We found that infection also increased numbers of BFU-E and CFU-E progenitor populations in the spleen and bone marrow and differentially altered erythroid gene expression in these organs. In the bone marrow, the mRNA expression of erythroid differentiation genes (α-globin, β-globin, ALAS2) were inhibited by 50%, but mRNA levels of erythroid receptor (c-kit, EpoR) and transcription factors (GATA1, GATA2, FOG1) were not affected by the infection. This suggests that infection has a negative effect on differentiation of erythroblasts. In the spleen, erythroid gene expression was enhanced by infection, indicating that the anemia activates a stress erythropoiesis response in the spleen. Analysis of cytokine mRNA levels in spleen and bone marrow found that IFN-γ mRNA is highly increased by *L. donovani* infection. Expression of the IFN-γ inducible cytokine, TNF-related apoptosis-inducing ligand (TRAIL), was also up-regulated. Since TRAIL induces erythroblasts apoptosis, apoptosis of bone marrow erythroblasts from infected hamsters was examined by flow cytometry. Percentage of erythroblasts that were apoptotic was significantly increased by *L. donovani* infection. Together, our results suggest that *L. donovani* infection inhibits erythropoiesis in the bone marrow by cytokine-mediated apoptosis of erythroblasts.

## Introduction

Visceral leishmaniasis (VL) is a parasitic disease caused by *Leishmania donovani* and related species (*L. infantum, L. chagasi)* which widely disseminate in the body by infecting and growing in phagocytes of the bone marrow, spleen, and liver [1]–[3]. Visceral leishmaniasis is characterized by massive hepatosplenomegaly, fever, anemia, and leucopenia [4]–[6]. The disease is usually fatal without drug treatment and even with drug treatment patients may die from the pathology or opportunistic bacterial infections [7], [8]. Visceral leishmaniasis caused by *L. donovani* is endemic in India, Bangladesh, Nepal, and Sudan [3]. *L. chagasi* causes leishmaniasis in South America and *L. chagasi* causes the disease in Southern Europe [3], where co-infection with HIV is prevalent [9], [10]. It is estimated that there are 500,000 reported new cases of VL each year [11].

Infection of mice with *L. donovani* has been widely used to study the immunology of VL. However, the course of the disease in mice greatly differs from that in man and the infection is not fatal in mice. Initially, there is parasite growth in the liver and spleen, but by 4–5 weeks the infection is resolved in the liver by a TH1 dependent granulomatous response [12]–[15]. The parasite persists as a chronic infection in the spleen with gradual destruction of spleen architecture [16]–[19]. Another experimental model of VL is the infection of Syrian golden hamsters (*Mesocricetus auratus)* with *L. donovani,* which has been used to study VL since the 1967 [20]. The disease in Syrian hamsters closely resembles human VL with relentless growth of parasites in the bone marrow, spleen, and liver, hepatosplenomegaly, anemia, leucopenia and death by 9–10 weeks after infection [21]–[25]. The study of cytokine responses of the hamster immune system during VL has been hindered by the lack of immunological reagents [26]. Recently, cytokine expression during VL in hamsters has been studied by measuring changes in cytokine mRNA levels [27], [28]. These studies show that the infection in hamsters progresses despite a strong TH-1 response characterized by high expression of IFN-γ mRNA in the spleen and bone marrow [28].

Few studies have examined how the parasite alters hematopoiesis and how these alterations contribute to the pathology associated with VL. Analysis of hematopoietic progenitors in the spleen and bone marrow of mice infected with *L. donovani* have demonstrated expansion of myeloid progenitor populations in the spleen and bone marrow of infected mice that is associated with increased levels of mRNA for colony stimulating factors GM-CSF, M-CSF, G-CSF [29]. Another study showed that *in vitro* co-culture of mouse bone marrow and spleen cells with a bone marrow derived macrophage cell line infected with *L. donovani* resulted in enhanced production of myeloid progenitors, which occurred through induction of GM-CSF and TNF-α by the infected macrophage cell line [30]. In the present study, we investigated the effects of *L. donovani* on erythropoiesis in the spleen and bone marrow of infected Syrian hamsters. We report that the numbers of erythroid progenitors are enhanced by infection in spleen and bone marrow, but erythroblast differentiation is differentially affected by the infection. In the bone marrow, erythroblast differentiation is inhibited with increased numbers of apoptotic erythroblasts, while in the spleen mRNA expression of erythroid-specific genes is enhanced, suggesting induction of a stress erythropoiesis response to the anemia, similar to that described in mice [31], [32].

## Results

### Infection of Hamsters with *L. donovani* Results in Severe Anemia

**Figure 1:**
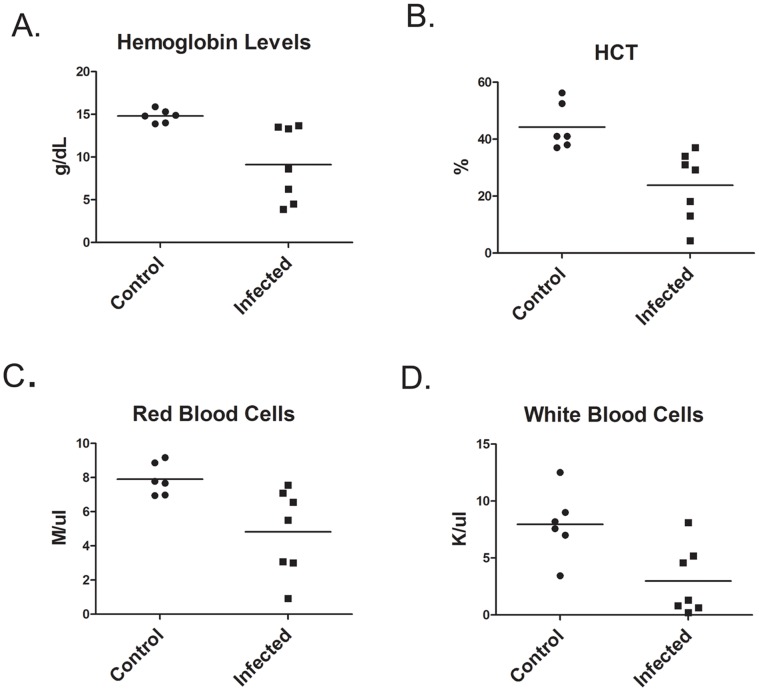
*L. donovani* infected hamsters develop severe anemia and leukopenia. Hematological analysis of blood from control hamsters and hamsters infected with *L. donovani* for 8 weeks. Data shown are from individual hamsters and the mean. Hemogloblin levels (A) and hematocrit (B) were statistically significant at p<0.01. Red blood cell (C) and white blood cell counts (D) were significant at p<0.05, Student’s t test.

**Figure 2:**
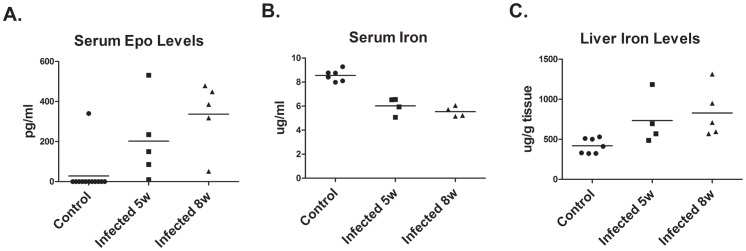
Erythropoietin and Iron levels in infected hamsters. (A) Serum erythropoietin (Epo) levels in control hamsters and hamsters infected with *L. donovani* for 5 and 8 were determined by ELISA. EPO levels were significantly increased by infection, p<0.005, one-way ANOVA. (B) Serum iron levels in control and infected hamsters were determined by ferrozone assay. Serum iron levels were significantly decreased by infection, p<0.0005, one-way ANOVA. (C). Liver iron levels in control and infected hamsters were determined by ferrozone assay. Liver iron levels were significantly increased by infection p<0.05, one-way ANOVA.

Hamsters infected with *L. donovani* develop a progressive disease in which uncontrolled parasite growth in the spleen and liver results in hepatosplenomegaly, anemia, leucopenia, and eventually death [21]–[24]. In the current study, hamsters were infected with 10^9^ amastigotes by intraperitoneal injection and disease assessed at 8 weeks post infection. Severe splenomegaly was present with a 20-fold increase in spleen weight (2.233±.326 g N = 4) compared to control non-infected hamsters (0.105±.0022 g N = 4). Hamsters were anemic with significantly decreased hemoglobin levels and hemocrit (**Figure 1**). Leucopenia was present with decreased numbers of red and white blood cells in the blood (**Figure 1** **).** Acute anemia results in a systemic stress response in which tissue hypoxia triggers production of erythropoietin by the kidney [33]. Therefore, circulating levels of Epo were determined by ELISA. Epo was detected in the serum of hamsters infected for 5–6 weeks and eight weeks, while Epo was only detected in the serum of one control non-infected hamster (**Figure 2**).

Since iron deficiency resulting from chronic infections can lead to anemia associated with chronic disease and inflammation, iron levels were measured in the blood and liver. Iron levels were decreased in the serum of infected hamsters and accumulated in the liver (**Figure 2**). The decrease in serum iron levels while statistically significant was relatively small and is not sufficient to account for the decreased numbers of circulating red blood cells. Further, there were no significant differences in MCV and MCHC between infected and control hamsters (data not shown), which would be expected to be decreased if iron deficiency is disrupting hemoglobin biosynthesis.

### *L. donovani* Infection Increases the Frequency of Erythroid Progenitors

**Figure 3:**
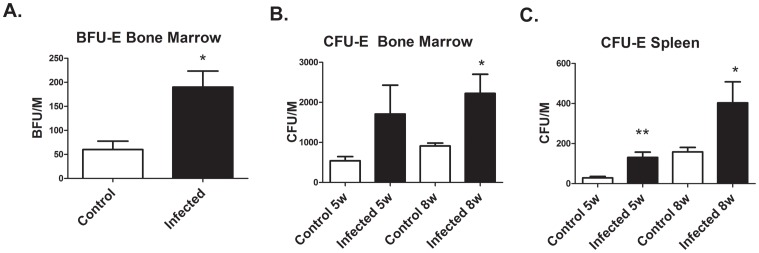
*L. donovani* infection increases BFU-E and CFU-E progenitor numbers in the bone marrow and spleen. (A) Frequency of BFU-E progenitors in the bone marrow of control hamsters and hamsters infected with *L. donovani* for 8 weeks was determined by the semi-solid colony assay. Data represent mean +/− SEM from hamsters. *p<0.05, Student’s t test. (B) and (C) Frequency of CFU-E progenitors in bone marrow and spleen from control and infected hamsters. Data represent mean +/− SEM from 3–5 hamsters. *p<0.05; *p<0.01, student’s t test.

To determine whether anemia associated with VL is due to reduction in erythroid progenitors, we determined the frequency of erythroid progenitors in the bone marrow and spleen by the generation of erythroid colonies in semisolid methylcellulose culture. To measure BFU-E progenitors, media was supplemented with Epo, SCF, and 10% hamster pokeweed mitogen spleen cell conditioned media. CFU-E progenitors were measured in media supplemented with Epo alone. Infection substantially increased the frequency of BFU-E and CFU-E progenitors in the bone marrow (**Figure 3** **)**. The frequency of CFU-E progenitors in the spleen was also increased, but numbers of CFU-E progenitors in the spleen of control and infected hamsters were less than in the bone marrow. These results indicate that there are sufficient numbers of erythroid progenitors in *Leishmania* infected hamsters to maintain erythropoiesis. Thus, disruption of erythropoiesis occurs during generation and differentiation of erythroblasts from CFU-E progenitors.

### Erythroid Gene Expression in the Bone Marrow and Spleen is Differentially Altered by *L. donovani* Infection

**Figure 4:**
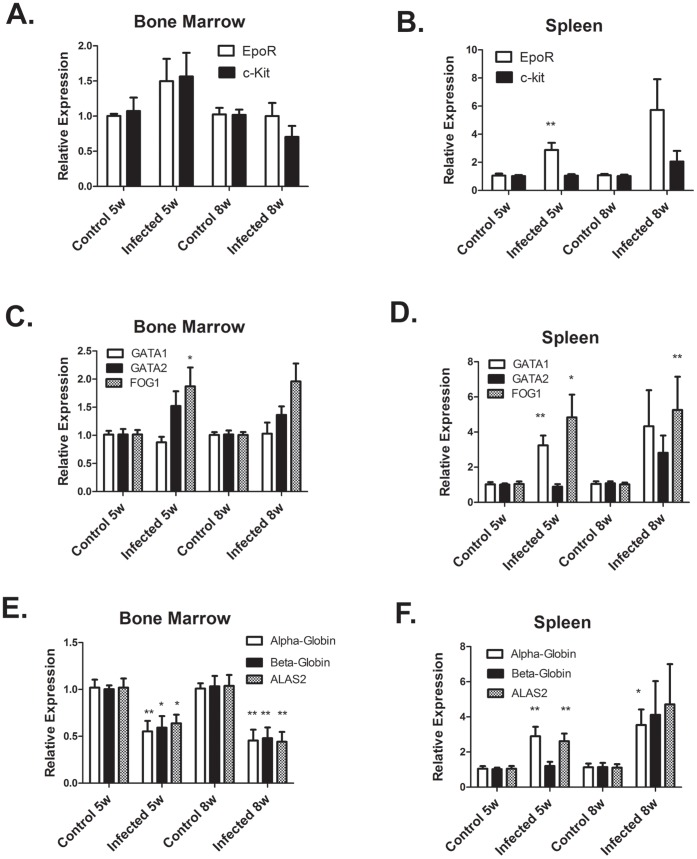
Erythroid gene expression is differentially altered by *L. donovani* infection in the bone marrow and spleen. mRNA levels of erythroid genes were determined by real-time RT-PCR in bone marrow (A,C,E) and spleen (B, D, F). mRNA levels were normalized to β-actin and expressed relative to control non-infected hamsters. Data represent mean +/− SEM of 6–8 hamsters in each group. *p<0.05; **p<0.01, Student’s t test.

We next examined the expression of erythroid genes in the bone marrow and spleen by real-time RT-PCR. We first cloned and sequenced cDNA segments of erythroid receptors c-kit and Epo-R; key transcription factors GATA1, GATA2, and FOG1; and erythroid differentiation genes β-globin and ALAS2. Real-time PCR primers were then designed from the sequences. We also designed primers for α-globin using sequence information in GenBank. In the bone marrow, expression of erythroid receptors and transcription factors mRNA were unchanged by *Leishmania* infection (**Figure 4**). However, erythroid differentiation genes α-globin, β-globin, and ALAS2 were inhibited by 50% in the bone marrow of *Leishmania* infected hamsters. In the spleen, expression of EpoR, GATA1, and FOG1 mRNA were increased by *Leishmania* infection at 5–6 weeks p.i (**Figure 4** **)**. Furthermore, levels of α-globin and ALAS2 transcripts were also increased by *Leishmania* infection. At 8 weeks p.i, expression of these genes also tended to be increased, but there was more variability in expression between individual infected hamsters (**Figure 4** **)**. We interpret these results to indicate that in the bone marrow a block in erythroid differentiation in *Leishmania* infected hamsters inhibits erythrocyte production. In the spleen, increased erythroid gene expression in *Leishmania* infected hamsters suggests that the spleen is responding to the anemia by activation of the stress erythropoiesis response that has been described in mice [31], [32].

### IFN-γ mRNA Levels in the Bone Marrow and Spleen are Highly Increased by *L. donovani* Infection

**Figure 5:**
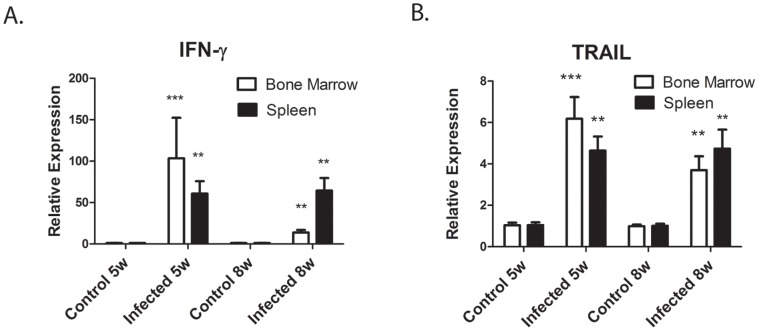
IFN-γ and TRAIL mRNA are highly expressed in *L. Donovani* infected hamsters. mRNA levels of IFN-γ (A) and TNF-related apoptosis inducing ligand (TRAIL) (B) were determined by real-time RT-PCR in the bone marrow and spleen of control non-infected hamsters and *L. donovani* infected hamsters. mRNA levels are expressed relative to control non-infected hamsters. Data represent mean +/−SEM of 6–8 hamsters in each group. **p<0.01; ***p<0.001, Student’s t test.

**Figure 6:**
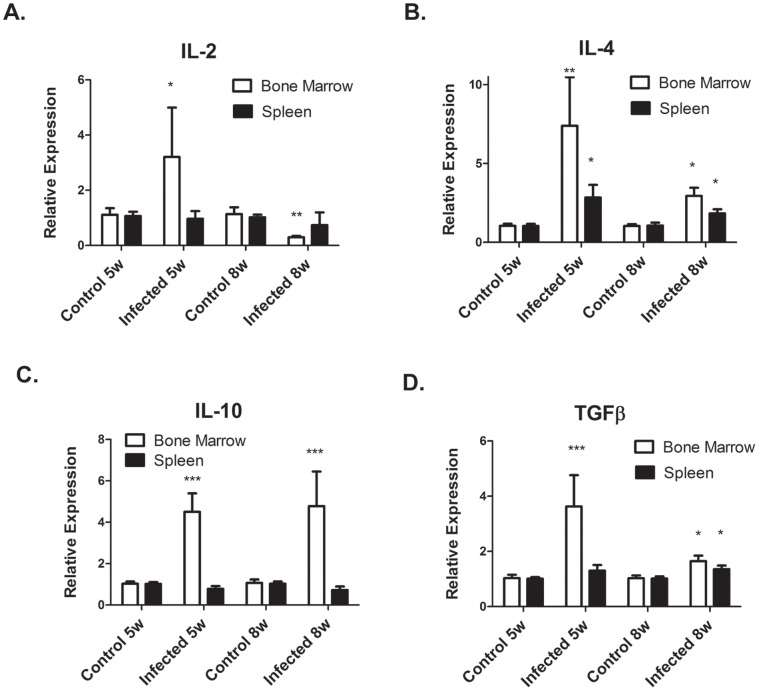
T cell and anti-inflammatory cytokine mRNA are increased by *L. donovani* infection. mRNA levels of T cell cytokines IL-2 and IL-4 (A and B) and anti- inflammatory cytokines IL-10 and TGF-β (C and D) in the spleen and bone marrow of control non-infected hamsters and *L. donovani* infected hamsters were determined by real-time RT-PCR, mRNA levels are expressed relative to control non-infected hamsters. Data represent mean +/− SEM of 6–8 hamsters in each group. *p<0.05; **p<0.01; ***p<.001, Student’s t test.

Since pro-inflammatory cytokines have an inhibitory effect on erythroid differentiation [34], we measured the expression of cytokine mRNA by real-time RT-PCR in control (non-infected) hamsters and hamster infected with *L. donovani* for 5–6 and 8 weeks. The most highly elevated cytokine was IFN-γ. IFN-γ mRNA expression was increased by 13–100 fold in both the bone marrow and spleen of infected hamsters (**Figure 5A** **)**. Thus, this result confirms previous studies which showed progression of VL in hamsters despite high expression of IFN-γ mRNA [28]. We also measured pro-inflammatory, T cell, and anti-inflammatory cytokine mRNA levels. There were no significant differences in pro-inflammatory cytokine (IL-1β, TNF-α, IL-6) mRNA levels between control and infected hamsters in the bone marrow or spleen (data not shown). T cell and anti-inflammatory cytokines in the bone marrow were increased at 5–6 weeks p.i. infection and declined at 8 week p.i. At 5–6 weeks, IL-2 and IL-4 were increased by 3–7 fold and IL-10 and TGF-β by 3–5 folds (**Figure 6** **)**. In the spleen, only IL-4 mRNA showed increased expression with infection (**Figure 6**). We also measured expression levels of IL-12p40 mRNA. Surprisingly, despite the increased expression of IFN-γ mRNA, IL-12 p40 mRNA expression levels between control and infected hamsters differed by less than 2-fold in both the spleen and bone marrow (data not shown).

### Expression of TRAIL mRNA is Increased by *L. donovani*

TRAIL (TNF-related apoptosis-inducing ligand) is a member of the TNF-family of cytokines that is induced by IFN-γ and is a negative regulator of erythropoiesis [35]–[37]. Trail mRNA expression was examined by real-time RT-PCR. To obtain sequence information to design real-time PCR primers, we first cloned and sequenced a cDNA segment of the hamster TRAIL gene. As shown in **Figure. 5B**, expression levels of TRAIL mRNA in the bone marrow and spleen were increased 3–6 fold by infection with *L. donovani.*

### Flow Cytometry Analysis of Erythroblasts

**Figure 7:**
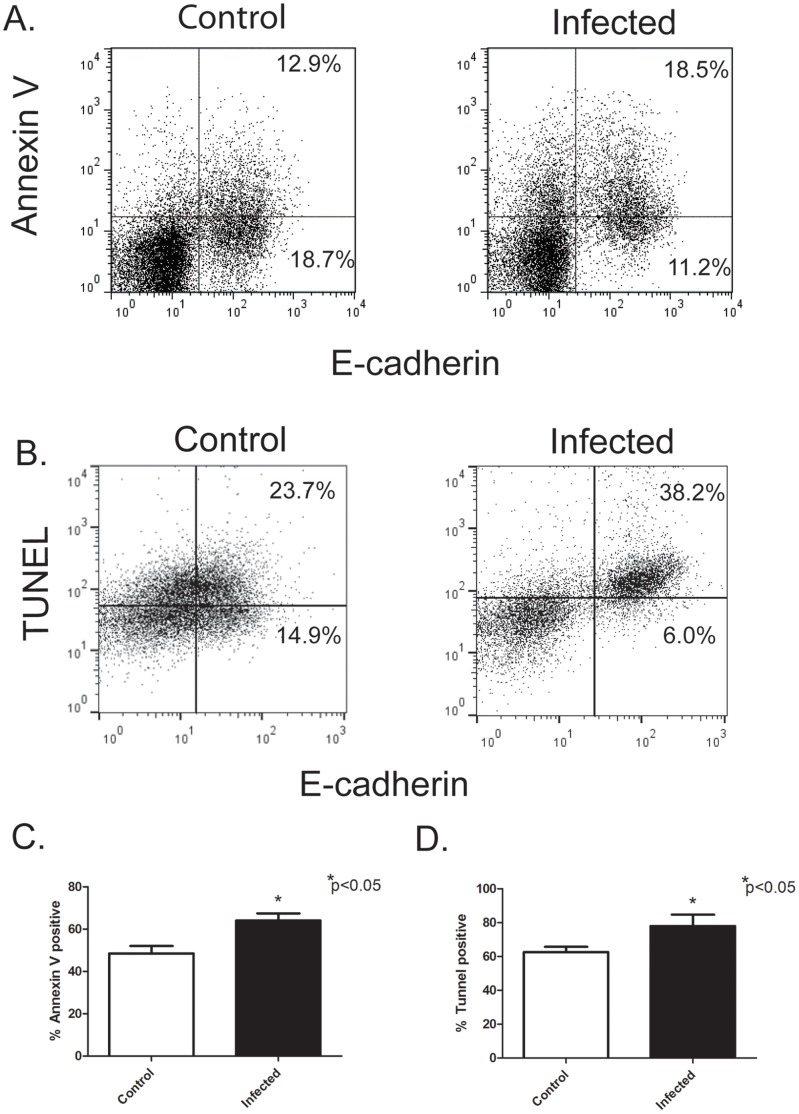
*L. donovani* infection increases apoptosis of erythroblasts. Apoptosis of bone marrow erythroblasts was examined by flow cytometry from control non-infected hamsters and *L. donovani* infected hamsters. Apoptosis was determined by Annexin-V binding (A) and the TUNEL Assay (B). Representative flow cytometry plots are from a control non-infected hamster and a hamster infected with *L. donovani* for 7 weeks. (A) Bone marrow cells were labeled with anti- E-cadherin antibody-e-Fluro 660 and FITC-Annexin V. Dead cells were excluded with 7-amino actinomycin (7-ADD). (B) BrdUTP incorporation by the TUNEL technique in erythroblasts was detected using Alexa-488 anti-BrdUTP and anti-E-cadherin-e-Fluro 660. (C) Percentage of E-cadherin positive erythroblasts that are Annexin-V positive. (D) Percentage of E-cadherin positive erythroblasts that are TUNEL positive. Data represent mean +/− SEM of 4 hamsters in each group. *p<0.05, Student’s t test.

Because TRAIL is a negative regulator of erythropoiesis and inducer of apoptosis, we hypothesized that the block in erythroid differentiation in *Leishmania* infected hamster results from apoptosis of erythroblasts during differentiation. To test this hypothesis, we determined if *Leishmania* infection increases the frequency of apoptotic erythroblasts by flow cytometry, using annexin-V binding and DNA fragmentation by the TUNEL assay. To detect hamster erythroblasts, a rat-monoclonal antibody (DECMA-1) that recognizes mouse, human, and canine E-cadherin (CD324) was used. E-cadherin is an epithelial cellular adhesion molecule that is also expressed on erythroblasts but not erythrocytes [38], [39]. It is also absent on all other hematopoietic cells. Bone marrow cells from hamsters infected with *Leishmania donovani* for 6–7 weeks and control non-infected hamsters were analyzed by flow cytometry for E-cadherin positive erythroblasts (**Figure 7** **)**. There were no significant differences in the % percentage of E-cadherin positive erythroblasts, 33% of bone marrow cells were E-cadherin positive for control hamsters and 31% for infected hamsters (data not shown)**.** However, the percentage of apoptotic erythroblasts that were annexin–V positive (**Figure 7A and C** **)** and TUNEL-positive (**Figure 7B and D** **)** were significantly higher in infected hamsters than control hamsters. Thus, *Leishmania donovani* infection in hamsters increases apoptosis of erythroblasts.

## Discussion

Visceral leishmaniasis (VL) in humans is a progressive disease that upon diagnosis can present a range of symptoms from asymptomatic infections, to sub-clinical infections, and active VL [40]–[42]. Experimental visceral leishmaniasis induced in Syrian Golden Hamsters by infection with *L. donovani* also progresses at different rates in individual hamsters. This is evident by the range in hemoglobin levels we observed in individual hamsters that ranged from levels near control hamsters to extremely low levels (below 5 g/dL) **(** **Fig. 1** **)**. Similar variability is also present in the HCT, red blood cells counts, and white blood cell counts. Syrian Golden Hamsters are out-bred, so genetic differences could be involved in the variability. Genetic factors may also influence progression of human infections with *L. donovani* or *L. chagasi* as there are large differences in the ratio of asymptomatic to symptomatic infection among different racial groups [43], [44].

*L. donovani* establishes infections by infecting and replicating in macrophages of bone marrow, spleen, and liver [1]–[3]. The progressive parasite burden in these organs is due in part to the ability of the parasite to alter hematopoiesis. *L. donovani* infection of mice results in expansion of progenitor cells and proliferative activity in the spleen and bone marrow [29]. This increase is highly selective for myleopoiesis. In the spleen, numbers of colony-forming unit-granulocyte, monocyte (CFU-GM) increased 20–30- fold at 28–56 days post-infection. Erythroid progenitors, burst-forming unit-erythrocyte (BFU-E), also increased, but to a lesser extent. Overall, splenomegaly was present, with 2–4 fold increase in total spleen cell numbers. An even greater selective expansion of hematopoiesis likely occurs in infected hamsters. For example, spleen weights in *L. donovani* infected hamsters increased 20-fold compared to control non-infected hamsters. Although we did not measure CFU-GM progenitor numbers, CFU-E progenitors in the spleen of infected hamsters were increased only 4-fold. A similar 4–5 fold increase in BFU-E and CFU-E progenitors were observed in bone marrow cells from infected hamsters.

The selective expansion of myleopoiesis during VL occurs together with alternations in erythropoiesis. Erythropoiesis in the bone marrow maintains the steady-state levels of circulating erythrocytes [45]. Thus, alternations in erythropoiesis in *L. Donovani* infected individuals contributes to a progressive decline in circulating erythrocytes and eventually anemia, which occurs in almost all VL patients. Analysis of bone marrow biopsies of VL patients indicate cellular hyperplasia with many abnormal erythroblasts, which include large erythroblasts containing giant lysosomes and nuclei with little condensed chromatin and multinuclear erythroblasts with irregular nuclei, and nuclear budding [46]–[48]. This dysplasia is similar to myelodysplasia observed in Myelodysplastic syndromes (MDS), which are stem cell disorders in which abnormal proliferation and differentiation of hematopoietic progenitor cells results in ineffective erythropoiesis [49], [50]. Erythrocytes that are produced during VL are also more susceptible to oxidative damage and have shortened lifespan [51]–[53].

The present study examined for the first time the expression of erythroid genes in the bone marrow during VL. Proliferation and differentiation of erythroid progenitors requires interactions of stem cell factor with its receptor (c-Kit) and eyrthropoietin with its receptor (EpoR) [54], [55]. Erythropoietin and EpoR are also required for survival and differentiation of erythroblasts [55]. Differentiation of erythroblasts requires expression of GATA1, GATA2 and FOG1 transcription factors [56]–[59]. We found that mRNA levels of c-Kit, EpoR-R, GATA1, GATA2, and FOG1 were not affected by *L. donovani* infection of hamsters. Thus, the expression of receptors and transcription factors required for erythropoiesis is not altered by VL. However, we did observe decreased expression of erythroid differentiation genes (alpha-globin, beta-globin, and ALAS2), which are target genes of GATA1. These results indicate a block in erythroblast differentiation could be due suppression of GATA1-induced expression of erythroid differentiation gene and/or through decreased numbers of erythroblasts.

Homeostasis of circulating erythrocytes is a balance between erythrocyte production by the bone marrow and red blood cell destruction. Apoptosis of immature erythroblasts is a key pathway by which bone marrow production of erythrocytes is regulated [reviewed in 45]. During normal physiological conditions, apoptosis acts as a negative control of the rate of maturation of immature erythroblasts. This occurs through the interaction of FasL and membrane TRAIL on mature erythroblasts with Fas and TRAIL receptors on immature erythroblasts, which activates the caspase cascade that cleaves GATA1 and triggers apoptosis [45]. As the erythroblasts mature, the erythroblasts become resistant to apoptosis induced by Fas cross-linking and lose expression of Trail receptors. During pathological conditions, up-regulation of Fas and FasL expression can exacerbate Fas-control of erythropoiesis. For example, Fas and FasL expression is increased in the bone marrow of MDS patients compared to controls [60]. Also, the death receptor ligand TRAIL and its receptors TRAILR1 and TRAILR2 are increased in bone marrow of MDS patients and hemopoietic progenitor cells from MDS patients are more sensitive to TRAIL-mediated apoptosis [61], [62]. Therefore, in the current study we examined apoptosis of bone marrow erythroblasts during *L. donovani* infection of hamsters by flow cytometry. To detect hamster erythroblasts, we used a rat monoclonal antibody to E-cadherin, which we found to cross-react with hamster. E-cadherin is expressed primarily by early immature erythroblasts and declines in expression as the erythroblast matures [38], [39]. Other antibodies used to detect erythroblasts, such as antibodies to mouse TER119 and human glycophorin A did not cross-react with hamster. We were also unable to find a cross-reactive antibody to CD71, which is used in flow cytometry in combination with antibodies to TER119 or glycophorin A to distinguish erythroblast stages. Our results do show that E-cadherin^+^ erythroblasts from *L. donovani*-infected hamsters have higher percentage of apoptotic cells, as detected by Annexin-V binding and the TUNEL assay, than erythroblasts from normal control hamsters. Thus, *L. donovani* infection of the bone marrow increases apoptosis during erythropoiesis.

In this study, we also examined erythroid gene expression in the spleen. In mice, erythropoiesis in the spleen occurs in response to anemia [32,32]. The response is regulated by high levels of circulating erythropoietin, which induces proliferation and differentiation of stress BFU-E progenitors into erythroblasts. Our data show that mRNA expression of erythroid genes is enhanced in *L.donovani-*infected hamsters. Expression levels tended to be higher at 8 weeks post-infection compared to 5 weeks, but there was also more variability in expression among individual hamsters, which likely reflects the variability in VL disease progression. We also observed higher levels of serum erythropoietin in *L. donovani* infected hamsters compared to control hamsters, which also tended to be higher at 8 weeks post-infection. The results are consistent with a stress erythropoiesis in infected hamsters induced by the anemia. Whether this stress erythropoiesis is countered by increased apoptosis of erythroblasts in the spleen of infected hamsters needs to be examined. However, erythropoietin has been shown in mice to inhibit apoptosis during the spleen stress erythropoiesis response by suppressing Fas-FasL expression [63].

The few studies that have examined mechanisms responsible for alterations in hematopoiesis focused on examining effects of cytokines and growth factors on the enhanced myelopoiesis during VL. Enhanced myelopoiesis in mice is associated with increased mRNA for growth factors GM-CSF, M-CSF, and G-CSF [29]. *L. donovani* infection of mouse bone marrow derived macrophages *in vitro* and co-incubation with spleen cells increased CFU-GM colony formation through the production of GM-CSF and TNF-α [30]. Mouse studies with TNF-α knockout mice have shown that TNF-α is required to control intracellular growth of *L. donovani* and promote granuloma formation [64], but is also responsible for remodeling the splenic marginal zone [18]. TNF-α may also alter erythropoiesis during VL, since TNF-α has been reported to inhibit erythropoiesis *in vitro* [34, 65, and 66]. However, we did not observe increased mRNA expression of TNF-α in the spleen or bone marrow of infected hamsters at 5 and 8 wks post-infection compared to control hamsters. This was not unexpected, since prior studies [27], [28] with hamsters have shown TNF-α expression in the spleen is induced early at 3–10 days post-infection and then declines. Whether TNF-α alters erythropoiesis early in the infection needs to be examined. However, its absence at 5 and 8 weeks post-infection suggest TNF-α is not involved in inhibiting erythropoiesis at these time points.

Our data show that mRNA for IL-10, TGF-β, and IFN-γ mRNA is up-regulated in bone marrow of *L. donovani* infection. Both IL-10 and TGF-β have been shown *in vitro* to inhibit erythropoiesis [67], [68]. IL-10 inhibits BFU-E growth by suppressing T cell production of GM-CSF [67]. Since we observed increased BFU-E and CFU-E progenitor numbers in infected hamster, this effect of IL-10 does not appear to be involved in the inhibiting erythropoiesis in infected hamsters. TGF-β acts by inducing cell cycle arrest of immature erythroblasts and accelerating differentiation [68]. TGF-β is also involved in T cell immunosuppression during VL in infected hamsters and induces lymphocyte apoptosis [69], [70]. Interestingly, while TGF-β induces lymphocyte apoptosis in hamsters, it does not appear to induce apoptosis in human erythroblasts. [68]. Whether TGF-β is a contributing factor to inhibition of erythropoiesis during VL needs further study.

The cytokine that we found to be induced in high amounts in *L. donovani* infected hamsters is IFN-γ. IFN-γ mRNA is produced in both the spleen and bone marrow throughout the course of infection in hamsters, with induction as early as 7–10 day post-infection [27], [28]. IFN-γ is also present in high amounts in the serum of human patients with active VL [71]–[73]. IFN-γ in *vitro* bone marrow studies suppresses proliferation and differentiation of erythroblasts [34, 74, and 75] and induces apoptosis in maturing erythroblasts [76]. Studies by Feli et al. [37] have shown that IFN-γ suppresses erythropoiesis by inducing expression of members of the TNF super family, including TRAIL, FASL (CD95L), and TWEAK, which interact with their receptors on target cells to induce apoptosis. Other studies have identified TRAIL as negative regulator of erythropoiesis [35, 36, 61, and 62]. In the current study, we showed that TRAIL mRNA expression in bone marrow and spleen is up-regulated by *L. donovani* infection. Thus, our studies are consistent with *L. donovani* infection inhibiting erythropoiesis by inducing macrophage expression of TRAIL, which then interacts with its receptors on immature erythroblasts to induce apoptosis. IFN-γ may also be involved in enhanced myelopoiesis, since IFN-γ has been show to expand early hematopoietic progenitor cell populations, resulting in a bias toward the myeloid lineage [77], [78].

## Materials and Methods

### Hamsters

Six −8 week old outbred Syrian golden hamsters (*Mesocricetus auratus*) were obtained from Charles River (Wilmington, DE). This study was carried out in strict accordance with the recommendations of Office of Laboratory Animal Welfare, National Institutes of Health. All procedures in this study were approved by the Institutional Animal Care and Use Committee of the Ohio State University, Columbus, Ohio.

### Parasites and Infection

*L. donovani* (LV82) was maintained in our laboratory by passage in Syrian golden hamsters. Hamsters were infected with 1×10^9^ *L. donovani* amastigotes by i.p. injection. Amastigotes for infection were obtained from the spleens of infected stock hamsters. The hamsters developed progressive illness resulting in death by 10 weeks. Groups of 3–5 infected hamsters and 5 control non-infected hamsters were euthanized at 5–6 weeks and 8 weeks post-infection. Blood, spleens, livers, and bone marrow were aseptically removed. The spleens were weighted and single cell suspensions prepared by gentle teasing. The femurs were isolated from surrounding tissue, cut at each end with scissors, and the bone marrow cells flushed from marrow cavity with Iscove’s modified Dulbecco’s medium (IMDM). Livers were snap frozen and stored −80°C.

### Hematological Analysis

Blood was collected from euthanized hamsters by cardiac puncture in heparinized tubes (Sigma) and submitted to the Hematology Lab of the Ohio State University Veterinary Hospital for hematological analysis. Blood was also collected in non-heparinized tubes and allowed to clot overnight at 4°C. Serum was obtained and stored at −80°C until analyzed for serum erythropoietin and iron levels. Serum erythropoietin levels were determined by ELISA using a mouse erythropoietin ELISA kit (R&D Systems, Minneapolis, MN) and mouse erythropoietin as standard.

### Serum and Liver Iron Analysis

Serum were de-proteinized by 1:1 dilution with 10% TCA in 1N HCL and incubation at 95° for 1 hr in microcentrifuge tubes. Liver tissue was weighted and 75–150 mg of tissue homogenized in 1 ml of molecular grade water using a tissue homogenizer. Liver homogenates were de-proteinized by1:1 dilution with 10% TCA in 1N HCL and heating at 65°C for 20 hrs. Precipitated proteins were removed by centrifugation and iron content determined by the ferrozone assay [79].

### Colony Assays for BFU-E and CFU-E

Single cell suspensions of splenocytes and bone marrow cells were incubated with RBC lysing buffer to lyse red blood cells, pelleted by centrifugation, resuspended in IMDM with 2% FBS and counted. For the CFU-E assay, 5×10^5^ bone marrow cells and 8×10^5^ splenocytes were plated in duplicate 35 mm culture plates containing methylcellulose media (Stem Cell Technologies) with 3 units/ml Epo. After 3 days of culture at 37°, the plates were stained with of 2, 7-diaminofluroene (DAF) to detect hemoglobin and hemoglobin positive colonies counted under an inverted microscope [80]. For the BFU-E assay, 3×10^5^ bone marrow cells were plated in duplicate 35 mm culture plates containing methylcellulose media with 3 units/ml Epo, 150 ng/ml mouse SCF, and 10% pokeweed mitogen hamster spleen conditioned media. After 10 days of culture, colonies of least 30 red-colored cells were counted.

### Flow Cytometry Analysis

Bone marrow cells were isolated from hamsters infected with *Leishmania donovani* and control non-infected hamsters. Cells were stained with E-cadherin (CD234) monoclonal antibody DECMA-1 conjugated with e-Fluro 660 antibody (e-Bioscience) washed in FACS buffer (phosphate-buffered saline [PBS], 0.1% NaN_3_, 1% bovine serum albumin [BSA]), and incubated with FITC-annexin-V and 7-amino-actinomycin D in annexin-V binding buffer (e-Bioscience) to evaluate apoptosis of erythroblasts. To indicate background staining, rat IgG1 isotype control e-Fluro 660 was used. Cells were analyzed using a FACSCalibur flow cytometer (Becton-Dickinson). Cell acquisition was obtained using the CellQuest software program (Becton-Dickinson) and the data analyzed by FlowJo software (Tree Star).

### TUNEL Assay

DNA fragmentation of erythroblasts from *Leishmania donovani* infected hamsters and control hamsters was determined using the APO-BrdU TUNEL assay kit (Life Technologies). The TUNEL assay was performed using bone marrow cells according to the manufacturer’s instructions and analyzed by flow cytometry using E-cadherin antibody conjugated with e-Fluro 660 and Alexa 488 anti-BrdU antibody.

### Cloning of Hamster cDNA and Real-time RT- PCR Primer Design

To obtain hamster gene sequences for primer design, we designed PCR primers from the mouse gene sequences that were conserved with rat and then used these primers to amplify hamster cDNA by PCR. PCR products 300–700 bp in size were isolated by agarose gel electrophoresis and purified on Qiagen spin columns (QIAquick gel extraction kit). PCR products were cloned into pGEM-T Easy Vector (Promega) and transformed into DH5α *E. coli.* (Invitrogen). Plasmid DNA was isolated from 2–3 colonies using Qiagen QIAprep Miniprep Kit and sequenced. Identity of the hamster cDNA sequences were confirmed by NCBI Blast search and were 82–93% identical to the mouse and rat homologues DNA sequences for GATA1 (JX569328), GATA2 (JX569329), FOG1 (JX569330), EpoR (JX569331), c-Kit (JX569332), β-globin (JX569333), ALAS2 (JX569334), and TRAIL (JX569335) have been submitted to GenBank. Primers (Table 1) for real-time RT-PCR were designed from these sequences using PrimerQuest (Integrated DNA Technologies). Hamster gene sequences in GenBank were used to design primers for α-globin (X57029), IL-6 (AB028635), IL12p40 (AB085792), and β-actin (AJ312092). Primer sequences for IL-1β, IL-2, IL-4, IL-10, IFN-γ, and TGF-β have been previously reported [81]–.

**Table 1:** List of primers used for RT-PCR.

Gene	Forward Primer	Reverse Primer	Product Size(bp)
IL-1β	TACAACAAGAGCTTCCGGCA	GGCCACAGGTATCTTGTCGT	355
IL-2	CCAGTGCCTGGAAGAAGAACTT	CATCTTCCAAGTGAAAGCTTTTGCT	147
IL-4	CCACGGAGAAAGACCTCATCTG	GGGTCACCTCATGTTGGAAATAA	72
IL-6	AGCCAGAGTCATTCAGAGCACCAT	AGGTTGGGCTAGGCGTGACTATTT	95
IL-10	TGGACAACATACTACTCACTG	GATGTCAAATTCATTCATGGC	308
IL-12p40	ACTCACGACTGCTGCTTCACAAGA	ACCGTCCAGAGTAATTTGCTGCCT	123
IFN-γ	GACAACCAGGCCATCC	CAAAACAGCACCGACT	226
TNFα	CGAGTGACAAGCCTGTAGCCCA	TGATGGCAGAGAGGAGGTTGA	262
TGF-β	GCGGCAGCTGTACATCGA	GGCTCGTGAATCCACTTCCA	57
β-Actin	TCCTGTGGCATCCACGAAACTACA	ACAGCACTGTGTTGGCATAGAGGT	88
α-globin	CACCACCAAGACCTACTTTCC	CAGTGGCTCAGGAGCTTGA	197
β-globin	TGCACGTGGATCCTGAGAACTTCA	AGCCACCACCTTATGAAAGGCAGA	174
ALAS2	ATCCCAAGCCAACCATTCCCTAGT	TAACCAAAGGCCTGGCTTCCTGTA	104
EpoR	TGAGTCACGAAAGTCATGTCGCCT	CATTGATGTGGATGATGCGGTGGT	167
c-Kit	TAAGTCAGATGCTGCCATGACGGT	TCATGTGATTGCCCAGGTAGCTCA	119
GATA1	ATACAAGCCACAGGCATTGCACAG	CCTGACTTTGCTGGGCCTTTCTTT	198
GATA2	AAGAAGTGTCTCCAGATCCCAGCA	TGCCACCTTCCATCTTCATGCTCT	144
FOG1	ACACCCTGTGAAGGAACCAGTAGA	ATGACTGCGGTAGCAAGGATGGAT	111
Trail	AGTATTCCTTTCCCGCCCAGAAGT	ATCGGAGCTAAGGCTGAACTGCTT	94

### Isolation of Hamster RNA and Real-time RT PCR Analysis

Spleen and bone marrow cells were lysed by homogenization with TRIzol reagent (Invitrogen) and RNA isolated using the RNeasy mini kit (Qiagen). Residual DNA was removed during RNA purification by on-column DNase digestion. RNA was reversed-transcribed using oligo (dT)_15_ primers by the Promega Reverse Transcription System. The mRNA expression was analyzed by real-time RT-PCR using FastStart DNA STBR Green 1 reaction mixture (Roche). The amplification conditions were 95°C for 10 minutes followed by 40 cycles of 95°C for 15 s, 57°C for 30 s, and 72°C for 30 s. Each measurement was carried out in duplicate. Relative expression was calculated by the ΔCT method using β-actin expression as the normalize [84]. Results are expressed as relative gene expression to samples from uninfected hamsters. PCR products were electrophoresed on 2% agarose gels to confirm amplification of products with the correct size.

### Statistical Analysis

Statistical analysis was performed using Prism 4 software (GraphPad Software, San Diego). One-way ANOVA was used for multiple comparisons. Pairwise comparisons were done using Student’s t-test.

## References

[1] Alexander J, Russell DG (1992) The interaction of Leishmania species with macrophages. Adv Parasitol 31: 175–254. 1496927 10.1016/s0065-308x(08)60022-6

[2] Liew FY, O’Donnell CA (1993) Immunology of leishmaniasis. Adv Parasitol 32: 161–258. 8237615 10.1016/s0065-308x(08)60208-0

[3] Murray HW, Berman JD, Davies CR, Saravia NG (2005) Advances in leishmaniasis. Lancet 366: 1561–1577. 16257344 10.1016/S0140-6736(05)67629-5

[4] Zijlstra EE, Ali MS, el-Hassan AM, el-Toum IA, Satti M, et al (1992) Clinical aspects of kala-azar in children from the Sudan: a comparison with the disease in adults. J Trop Pediat 38: 17–21. 10.1093/tropej/38.1.17 1315397

[5] al-Jurayyan NA, Al-Naaer M, Al-Fawaz I, al-Herbish AS, al-Mazrou AM, et al (1995) The haematological manifestations of visceral leishmaniasis in infancy and childhood. J Trop Pediat 41: 143–148. 10.1093/tropej/41.3.143 7636932

[6] Pearson RD, Sousa AQ (1996) Clinical Spectrum of leishmaniasis. Clin Infect Dis 22: 1–13. 8824958 10.1093/clinids/22.1.1

[7] Santos MA, Marques RC, Fairas CA, Vasconcelos DM, Stewart JM, et al (2002) Predictors of an unsatisfactory responses to pentavalent antimony in the treatment of American visceral leishmaniasis. Rev Soc Bras Med Trop 35: 629–633. 12612746 10.1590/s0037-86822002000600014

[8] de Araujo VE, Morais MH, Reis IA, Rabello A, Carnerio M (2012) Early clinical manifestions associated with death from visceral leishmaniasis. Plos Neg Trop Dis 6: e1511. 10.1371/journal.pntd.0001511 PMC3274500 22347514

[9] Pasaquau F, Ena J, Sanchez R, Cuadrado JM, Amador C, et al (2005) Leishmaniasis as an opportunistic infection in HIV-infected patients: determinants of relapse and mortality in a collaborative study of 228 episodes in a Mediterreanean region. Eur J Clin Microbiol Infect Dis 24: 411–418. 15928908 10.1007/s10096-005-1342-6

[10] Alvar J. Aparicio P, Aseffa A, Den Boer M, Canavate C, et al (2008) The relationship between Leishmaniasis and AIDS: the second 10 years. Clin Microbiol Rev 21: 334–359. 18400800 10.1128/CMR.00061-07 PMC2292576

[11] Savioli L, Daumerie D, Crompton DWT, Annon. (2010) Leishmaniasis. In: Savioli L, Daumerie D, Crompton DWT, editors. Working to overcome the global impact of neglected tropical diseases. First WHO report on neglected tropical diseases. Geneva: World Health Organization. 91–96.

[12] Miralles GD, Stoeckle MY, McDermott DF, Finkelman FD, Murray HW (1994) Th1 and Th2 cell-associated cytokines in experimental visceral leishmaniasis. Infect Immun 62: 1058–1063. 8112840 10.1128/iai.62.3.1058-1063.1994 PMC186224

[13] Wilson ME, Sandor M, Blum AM, Young BM, Metwali A, et al (1996) Local suppression of IFN-γ in hematic granulomas correlates with tissue-specific replication of *Leishmania chagasi.* . J Immunol 156: 2231–2239. 8690913

[14] Engwerda CR, Murphy M, Cotterell SE, Smelt SC, Kaye PM (1998) Neutralization of IL-12 demonstrates the existence of discrete organ-specific phases in the control of *Leishmania donovani* . Eur J Immunol 28: 669–680. 9521077 10.1002/(SICI)1521-4141(199802)28:02<669::AID-IMMU669>3.0.CO;2-N

[15] Engwerda CR, Kaye PM (2000) Organ-specific immune responses associated with infectious disease. Immunology Today 21: 73–78. 10652464 10.1016/s0167-5699(99)01549-2

[16] Smelt SC, Engwerda CR, McCrossen M, Kaye PM (1997) Destruction of follicular dendritic cells during chronic visceral leishmaniasis. J. Immunol. 158: 3813–3821. 9103448

[17] Melby PC, Tabares A, Restrepo BJ, Cardona AE, McGuff HS, et al (2001) *Leishmania donovani: e*volution and architecture of the splenic cellular immune response related to control of infection. Exp Parasitol 99: 17–25. 11708830 10.1006/expr.2001.4640

[18] Engwerda CR, Ato M, Cotterell SE, Mynott TL, Tschannerl A, et al (2002) A role for tumor necrosis factor-α in remodeling the splenic marginal zone during *Leishmania donovani* infection. Am J Path 161: 429–437. 12163368 10.1016/s0002-9440(10)64199-5 PMC1850733

[19] Engwerda CR, Ato M, Kaye PM (2004) Macrophages, pathology, and parasite persistence in experimental visceral leishmaniasis. Trends Parasitol 20: 524–530. 15471704 10.1016/j.pt.2004.08.009

[20] Ott KJ, Hanson WL, Stauber L (1967) Course of infection of *Leishmania donovani.* . J Parasitol 53: 641–645. 6026857

[21] Farrell JP (1976) *Leishmania donovani*: acquired resistance to visceral leishmaniasis in the golden hamster. Exp Parasitol 40: 89–94. 950004 10.1016/0014-4894(76)90069-2

[22] Gifawesen C, Farrell JP (1989) Comparison of T-cell responses in self-limiting versus progressive visceral *Leishmania donovani* infections in golden hamsters. *Infect Immun* 57: 3091–3096. 2528507 10.1128/iai.57.10.3091-3096.1989 PMC260774

[23] Evans TG, Smith D, Pearson RD (1990) Humoral factors and nonspecific immune suppression in Syrian hamsters infected with *Leishmania donovani.* . J Parasitol 76: 212–217. 2319422

[24] Biswas T, Chakraborty M, Naskar K, Ghosh DK, Ghosal J (1992) Anemia in experimental visceral leishmaniasis in hamsters. J Parsitol 78: 140–142. 1310731

[25] Requena JM, Soto M, Doria MD, Alonso C (2000) Immune and clinical parameters associated with *Leishmania infantum* in the golden hamster model. Vet Immunol Immunopathol 76: 269–281. 11044559 10.1016/s0165-2427(00)00221-x

[26] Zivcec M, Safronetz D, Haddock E, Feldmann H, Ebihara H (2011) Validation of assays to monitor immune responses in the Syrian golden hamster (*Mesocricetus auratus*). J Immunol Methods 368: 24–35. 21334343 10.1016/j.jim.2011.02.004 PMC3085612

[27] Melby PC, Tryon VV, Chandrasekar B, Freeman GL (1998) Cloning of Syrian hamster (*Mesocrietus auratus*) cytokine cDNAs and analysis of cytokine mRNA in experimental visceral leishmaniasis. Infect Immun 66: 2135–2142. 9573100 10.1128/iai.66.5.2135-2142.1998 PMC108174

[28] Melby PC, Chandrasekar B, Zhao W, Coe JE (2001) The hamster as a model of human visceral leishmaniasis: progressive disease and impaired generation of nitric oxide in the face of a prominent TH1-like cytokine response. J Immunol 166: 1912–1920. 11160239 10.4049/jimmunol.166.3.1912

[29] Cotterell SE, Engwerda CR, Kaye PM (2000) Enhanced hematopoietic activity accompanies parasite expansion in the spleen and bone marrow of mice infected with *Leishmania donovani.* . Infect Immun 68: 1840–1848. 10722572 10.1128/iai.68.4.1840-1848.2000 PMC97356

[30] Cotterell SE, Engwerda CR, Kaye PM (2000) *Leishmania donovani* infection of bone marrow stromal macrophages selectively enhances myelopoiesis, by a mechanism involving GM-CSF and TNF-α. Blood 95: 1642–1651. 10688819

[31] Perry J, Harandi OF, Paulson R (2007) BMP4, SCF, and Hypoxia cooperatively regulate the expansion of murine stress erythroid progenitors. Blood 109: 4494–4502. 17284534 10.1182/blood-2006-04-016154 PMC1885504

[32] Perry JM, Harandi OF, Porayette P, Hedge S, Kannan AK, et al (2009) Maintenance of the BMP4 dependent stress erythropoiesis pathway in the murine spleen requires hedgehog signaling. Blood 113: 911–918. 18927434 10.1182/blood-2008-03-147892 PMC2630276

[33] Sasaki R, Masuda S, Nago M (2000) Erythropoietin: multiple physiological functions and regulation of biosynthesis. Biosci Biotechnol Biochem 64: 1775–1793. 11055378 10.1271/bbb.64.1775

[34] Morceau F, Dicato M, Diecherich M (2009) Pro-inflammatory cytokine-mediated anemia: Regarding molecular mechanisms of erythropoiesis. Mediators Inflamm 2009: 405016. 20204172 10.1155/2009/405016 PMC2830572

[35] Zauli G, Secchiero P (2006) The role of the TRAIL/TRAIL receptors system in hematopoiesis and endothelial cell biology. Cytokine Growth Factor Rev 17: 245–257. 16750931 10.1016/j.cytogfr.2006.04.002

[36] Silvestris F, Cafforio P, Tucci M, Dammacco F (2002) Negative regulation of erythroblast maturation by Fas-L^+^/TRAIL^+^ highly malignant plasma cells: a major pathogenetic mechanism of anemia in multiple myeloma. Blood 99: 1305–1313. 11830480 10.1182/blood.v99.4.1305

[37] Felli N, Pedini F, Zeuner A, Petrucci E, Testa U, et al (2005) Multiple members of the TNF superfamily contribute to IFN-γ-mediated inhibition of erythropoiesis. J Immunol 175: 1464–1472. 16034083 10.4049/jimmunol.175.3.1464

[38] Armeanu S, Buhring HJ, Reuss-Borst M, Muller CA, Klein G (1995) E-cadherin is functionally involved in the maturation of the erythroid lineage. J Cell Biol 131: 243–249. 7559781 10.1083/jcb.131.1.243 PMC2120602

[39] Lammers R, Giesert C, Grunebach F, Marxer A, Vogel W, et al (2002) Monoclonal antibody 9C4 recognizes epithelial cellular adhesion molecule, a cell surface antigen expressed in early steps of erythropoiesis. Exp Hematol 30: 537–545. 12063020 10.1016/s0301-472x(02)00798-1

[40] Badaro R, Jones TC, Carvalho EM, Sampaio D, Reed SG, et al (1986) New perspectives on a subclinical form of visceral leishmaniasis. J Infect Dis 154: 1003–1011. 3782864 10.1093/infdis/154.6.1003

[41] Badaro R, Jones TC, Lorenco R, Cerf BJ, Sampaio D, et al (1986) A prospective study of visceral leishmaniasis in an endemic area of Brazil. J Infect Dis 154: 639–649. 3745974 10.1093/infdis/154.4.639

[42] Wilson ME, Jeronimo SM, Pearson RD (2005) Immunopathogenesis of infection with the visceralizing *Leishmania* species. Microb Patho 38: 147–160. 10.1016/j.micpath.2004.11.002 15797810

[43] E-l Hassan AM, Zijlstra EE, Ismael A, Ghalib HW (1995) Recent observations on the epidemiology of kala-azar in the eastern and central states of Sudan. Trop Geogr Med 47: 151–157. 8560585

[44] Ibrahim ME, Lambson B, Yousif AO, Deifalla N, Alnaiem DA, et al (1999) Kala-azar in a high transmission foci: an ethnic and geographical dimension. AM J Trop Med Hyg 61: 941–944. 10674674 10.4269/ajtmh.1999.61.941

[45] Testa U (2004) Apoptotic mechanisms in the control of erythropoiesis. Leukemia 18: 1176–1199. 15208642 10.1038/sj.leu.2403383

[46] Wickramasinghe SN, Abdalla SH, Kasili EG (1987) Ultrastructure of bone marrow in patients with visceral leishmaniasis. J Clin Pathol 40: 267–275. 3558859 10.1136/jcp.40.3.267 PMC1140897

[47] Yarali N, Fisgin T, Duru F, Kara A (2002) Myelodysplastic features in visceral leishmaniasis. Am J Hematol 71: 191–205. 12410574 10.1002/ajh.10200

[48] Kumar PV, Vasei M, Sadeghipour A, Sadeghi E, Soleimanpour H, et al (2007) Visceral leishmaniasis: bone marrow biopsy findings. J Pediatr Hematol Oncol 29: 77–80. 17279002 10.1097/MPH.0b013e31803076a8

[49] Hasle H (1994) Myelodysplastic syndromes in childhood-classification, epidemiology, and treatment. Leuk Lymphoma 13: 11–16. 10.3109/10428199409051647 8025513

[50] Hofmann WK, Ottmann OG, Ganser A, Hoelzer D (1996) Myelodysplastic syndromes: clinical features. Semin Hematol 33: 177–185. 8819228

[51] Sen G, Mukhopadhyay R, Ghosal J, Biswas T (2001) Oxidative damage of erythrocytes: a possible mechanism for premature hemolysis in experimental visceral lesihmaniasis in hamsters. Ann Hematol 80: 32–37. 11233773 10.1007/s002770000240

[52] Sen G, Mukhopadhyay R, Ghosal J, Biswas T (2004) Combination of ascorbate and α-tocopherol as a preventive therapy against structural and functional defects of erythrocytes in visceral leishmaniasis. Free Radic Res 38: 527–534. 15293561 10.1080/10715160410001665253

[53] Saha Roy S, Chowdhury KD, Sen G, Biswas T (2009) Oxidation of hemoglobin and redistribution of band 3 promote erythrophagocytosis in visceral leishmaniasis. Mol Cell Biochem 321: 53–63. 18777164 10.1007/s11010-008-9909-z

[54] Wu H, Klingmuller U, Acurio A, Hsiao JG, Lodish HF (1997) Functional interaction of erythropoietin and stem cell factor receptors is essential for erythroid colony formation. Proc Natl Acad Sci U S A 94: 1806–1810. 9050860 10.1073/pnas.94.5.1806 PMC19998

[55] Munugalavadla V, Kapur R (2005) Role of c-Kit and erythropoietin receptor in erythropoiesis. Crit Rev Onc/Hematol 54: 63–75. 10.1016/j.critrevonc.2004.11.005 15780908

[56] Leonard M, Brice M, Engel JD, Papayannopoulou T (1993) Dynamics of GATA transcription factor expression during erythroid differentiation. Blood 82: 1071–1079. 8353273

[57] Tsang AP, Fujiwara Y, Horn DB, Orkin SH (1998) Failure of megakaryopoiesis and arrested erythropoiesis in mice lacking the GATA-1 transcriptional cofactor FOG. Genes Dev 12: 1176–1188. 9553047 10.1101/gad.12.8.1176 PMC316724

[58] Ohneda K, Yamamoto M (2002) Roles of hematopoietic transcription factors GATA-1 and GATA-2 in the development of the red blood cell lineage. Acta Haematol 108: 237–245. 12432220 10.1159/000065660

[59] Ferreira R, Ohneda K, Yamamoto M, Philipsen S (2005) GATA1 function, a paradigm for transcription factors in hematopoiesis. Mol Cell Biol 25: 1215–1227. 15684376 10.1128/MCB.25.4.1215-1227.2005 PMC548021

[60] Claessens YE, Bouscary D, Dupont JM, Picard F, Melle J, et al (2002) *In vitro* proliferation and differentiation of erythroid progenitors from patients with myelodysplastic syndromes: evidence for Fas-dependent apoptosis. Blood 99: 1594–1601. 11861273 10.1182/blood.v99.5.1594

[61] Zang Y, Goodwin RG, Loken MR, Bryant E, Deeg HJ, et al (2001) Expression of tumor necrosis factor-related apoptosis-inducing ligand, Apo2L, and its receptors in myelodysplastic syndrome: effects on *in vitro* hemopoiesis. Blood 98: 3058–3065. 11698291 10.1182/blood.v98.10.3058

[62] Campioni D, Secchiero P, Corallini F, Melloni E, Capitani S, et al (2005) Evidence for a role of TNF-related apoptosis-inducing ligand (TRAIL) in the anemia of myelodysplastic syndromes. Am J Pathol 166: 557–563. 15681838 10.1016/S0002-9440(10)62277-8 PMC1602326

[63] Liu Y, Pop R, Sadegh C, Brugnara C, Haase VH, et al (2006) Supression of Fas-FasL coexpression by erythropoietin mediates erythroblast expansion during the erythropoietic stress response *in vivo.* . Blood 108: 123–133. 16527892 10.1182/blood-2005-11-4458 PMC1895827

[64] Murray HW, Jungbluth A, Ritter E, Montelibano C, Marino MW (2000) Visceral leishmaniais in mice devoid of tumor necrosis factor and response to treatment. Infect Immun 68: 6289–6293. 11035737 10.1128/iai.68.11.6289-6293.2000 PMC97711

[65] Dufour C, Corcione A, Svahn J, Haupt R, Poggi V, et al (2003) TNF-α and IFN-γ are overexpressed in the bone marrow of Fanconi anemia patients and TNFα suppresses erythropoiesis *in vitro.* . Blood 102: 2053–2059. 12750172 10.1182/blood-2003-01-0114

[66] Buck I, Morcau F, Cristofanon S, Heintz C, Chateauvieux S, et al (2008) Tumor necrosis factor α inhibits erythroid differentiation in human erythropoietin-dependent cells involving p38 MAPK pathway, GATA-1 and Fog-1 downregulaton and GATA-2 upregulation Biochem Pharm. 76: 1229–1239. 10.1016/j.bcp.2008.08.025 18805401

[67] Oehler L, Kollars M, Bohle B, Berer A, Reiter E, et al (1999) Interleukin-10 inhibits burst-forming unit-erythroid growth by suppression of endogenous granulocyte-macrophage colony-stimulating factor production from T cells. Exp Hematol 27: 217–223. 10029159 10.1016/s0301-472x(98)00049-6

[68] Zermati Y, Fichselson S, Valensi F, Freyssinier JM, Rouyer-Fessard P, et al (2000) Transforming growth factor inhibits erythropoiesis by blocking proliferation and accelerating differentiation of erythroid progenitors. Exp Hematol 28: 885–894. 10989189 10.1016/s0301-472x(00)00488-4

[69] Rodrigues V Jr, Santana da Silva J, Campos-Neto A (1998) Transforming growth factor β and immunosuppression in experimental visceral leishmaniasis. Infect Immun 66: 1233–1236. 9488418 10.1128/iai.66.3.1233-1236.1998 PMC108038

[70] Banerjee R, Kumar S, Sen A, Mookerjee A, Mukherjee P, et al (2010) TGF-β-regulated tyrosine phosphatases induce lymphocyte apoptosis in *Leishmania donovani*-infected hamsters. Immunol Cell Biol 89: 466–474. 20856262 10.1038/icb.2010.108

[71] Hailu A, van der Poll T, Berhe N, Kager PA (2004) Elevated plasma levels of interferon (IFN)-γ, IFN-γ inducing cytokines, and IFN-γ inducible CXC chemokines in visceral leishmaniasis. Am J Trop Med Hyg 71: 561–567. 15569785

[72] Cadlas A, Favali C, Aquino D, Vinhas V, van Weyenbergh J, et al (2005) Balance of IL-10 and Interferon-γ plasma levels in human visceral leishmaniasis: implications in the pathogenesis. BMC Infec Dis 5: 113. 16364177 10.1186/1471-2334-5-113 PMC1343567

[73] Ansari NA, Saluja S, Salotra P (2006) Elevated levels of interferon-gamma, interleukin-10, and interleukin-6 during active disease in Indian kala azar. Clin Immunol 119: 339–345. 16540374 10.1016/j.clim.2006.01.017

[74] Mamus SW, Beck-Schroeder S, Zanjani ED (1985) Suppression of normal human erythropoiesis by gamma interferon *in vitro*. Role of monocytes and T lymphocytes. J Clin Invest 75: 1496–1503. 3923039 10.1172/JCI111853 PMC425488

[75] Selleri C, Maciejewski JP, Sato T, Young NS (1996) Interferon-gamma constitutively expressed in the stromal microenvironment of human marrow cultures mediates potent hematopoietic inhibition. Blood 87: 4149–4157. 8639773

[76] Dai C, Krantz SB (1999) Interferon gamma induces upregulation and activation of caspases 1,3 and 8 to produce apoptosis in human erythroid progenitor cells. Blood 93: 3309–3316. 10233883

[77] Caux C, Moreau I, Saeland S, Bancherau J (1992) Interferon-γ enhances factor-dependent myeloid proliferation of human CD34^+^ hematopoietic cells. Blood 79: 2628–2635. 1375107

[78] Zhao X, Ren G, Liang L, Ai PZ, Zheng B, et al (2010) Brief Report: Interferon-γ induces expransion of Lin^-^Sca-1^+^C-Kit^+^ cells. Stem Cells 28: 122–126. 19890981 10.1002/stem.252

[79] Rebouche CJ, Wilcox CL, Widness JA (2004) Microanalysis of non-heme iron in animal tissues. J Biochem Biophys Methods. 58: 239–51. 10.1016/j.jbbm.2003.11.003 15026210

[80] Worthington RE (1987) Quantitation of erythroid differentiation in vitro using a sensitive colorimetric assay for hemoglobin. Exp Hematol 15: 85–92. 3490994

[81] Pacheco-Yepez J, Galvan-Moroyoqui JM, Meza I, Tsutsumi V, Shibayama M (2011) Expression of cytokines and their regulation during amoebic liver abscess development. Parasite Immunol. 33: 56–64. 10.1111/j.1365-3024.2010.01252.x 21155843

[82] Rama Iniguez S, Dea-Ayuela MA, Sanchez-Brunete JA, Torrado JJ, Alunda JM, et al (2006) Real-time reverse transcription-PCR quantification of cytokine mRNA expression in golden Syrian hamster infected with *Leishmania infantum* and treated with a new amphotericin B formulation. Antimicrob Agents Chemother 50: 1195–1201. 16569829 10.1128/AAC.50.4.1195-1201.2006 PMC1426985

[83] Li G, Duan T, Wu X, Tesh RB, Soong L, et al (2008) Yellow fever virus infection in Syrian golden hamsters: relationship between cytokine expression and pathological changes. Int J Clin Exp Pathol 1: 169–179. 18784801 PMC2480551

[84] Livak KJ, Schmittgen DT (2001) Analysis of relative gene expression data using real-time quantitative PCR and the 2^−ΔΔCt^ method. Methods 25: 402–408. 11846609 10.1006/meth.2001.1262

